# Supplementary material for: A yeast two-hybrid system to obtain triple-helical ligands from combinatorial random peptide libraries
Source: J Biol Chem. 2024 Sep 19;300(11):107794. doi: 10.1016/j.jbc.2024.107794 (PMC11533527; doi:10.1016/j.jbc.2024.107794)
Supplement: Supporting Figures and Tables [file mmc1.docx]

**Supporting Information**

**A yeast two-hybrid system to obtain triple-helical ligands from combinatorial random peptide libraries**

Ryo Masuda, Khine Phyu Phyu Thant, Kazuki Kawahara, Hiroya Oki, Tetsuya Kadonosono, Yuji Kobayashi, Takaki Koide

Table S1. Collagen-binding proteins and the identified binding sequences in human collagen

| Protein | Collagen-binding domain | Binding sequence^a,b^ | Ref. |
| --- | --- | --- | --- |
| PEDF | single-domain protein | KGHRGFSGL [α1(I)(87–95)/α1(I)(930–938)] | 1 |
| integrin α1β1  integrin α2β1  integrin α10β1  integrin α11β1 | αI domain  αI domain  αI domain  αI domain | GLOGEN [α1(II)(121–126)] ^c^  GFOGER [α1(I)(502–507)/α1(III)(322–327)] ^c^  GLOGEN [α1(II)(121–126)] ^c^  GFOGER [α1(I)(502–507)/α1(III)(322–327)] ^c^ | 2  3  4  5 |
| VWF  SPARC  DDR1/2,  aegyptin | A3 domain  EC domain  DS domain  -^d^ | RGQOGVMGF [α1(III)(405–413)]  GVMGFO [α1(III)(409–414)]  RGQOGVMGFO [α1(III)(405–414)]  RGQOGVMGF [α1(III)(405–413)] | 6  7  8,9  10 |
| MMP-1 | cat domain | OGPQGLAGQR [α1(II)(771–780)] | 11 |
| MMP-3 | cat domain | GAAGFOGAR [α1(III)(533–541)] | 12 |

^a^ Text in parentheses indicates the locations in collagen

^b^ “O” means 4-hydroxy-L-proline

^c^ The most potent sequence in collagen

^d^ Not reported


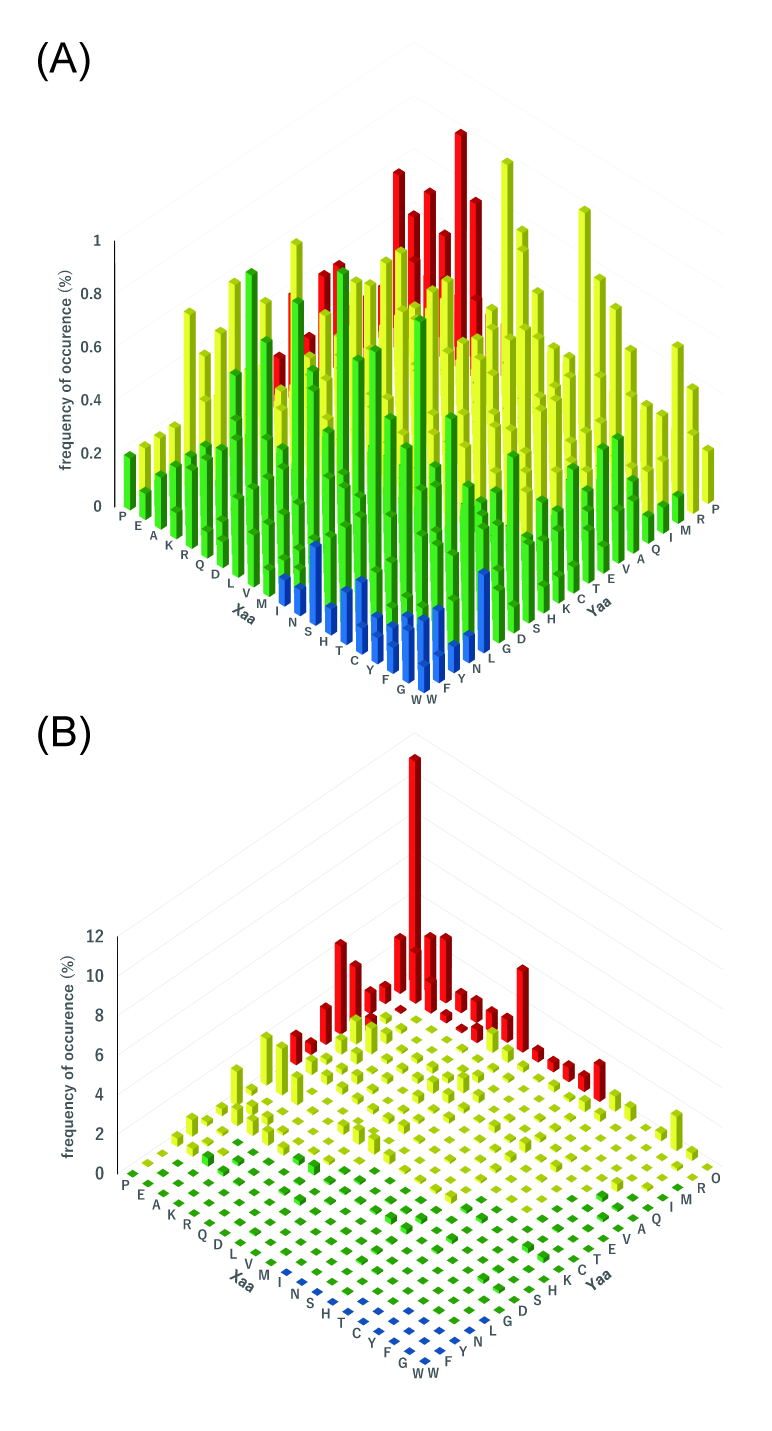


Fig. S1. Distribution of Gly-Xaa-Yaa triplets. (A) Theoretical distribution of triplets in which Xaa and Yaa are encoded by NNK codons. (B) A reduced view of Gly-Xaa-Yaa triplet distribution of human fibrillar collagen. Regarding the change from Fig. 2B to this figure, the maximum value of the Z-axis changes from 1% to 12%.


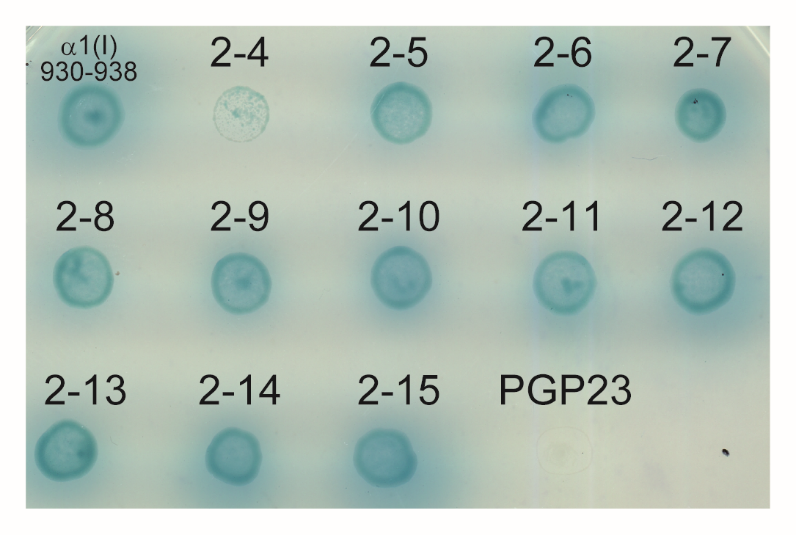


Fig. S2. Interaction of the AD-fusion peptides with the sequences obtained from the second selection with BD-fusion PEDF in yeast cells. PGP23 is GAL4-AD-fusion (PGP)23. The cells were cultured at 25°C for 5 days.

**pepα1(I)930-938**

H-Tyr-(Pro-Hyp-Gly)_4_-Pro-Lys-Gly-His-Arg-Gly-Phe-Ser-Gly-Leu-Hyp-Gly-(Pro-Hyp-Gly)_4_-Pro-NH_2_

T_R_: 14.0 min

**pep3**

H-Tyr-(Pro-Hyp-Gly)_4_-Pro-Arg-Gly-His-Arg-Gly-Phe-Leu-Gly-Leu-Hyp-Gly-(Pro-Hyp-Gly)_4_-Pro-NH_2_

T_R_: 17.7 min

**pep4**

H-Tyr-(Pro-Hyp-Gly)_4_-Pro-Arg-Gly-Ala-Arg-Gly-Leu-His-Gly-Leu-Hyp-Gly-(Pro-Hyp-Gly)_4_-Pro-NH_2_

T_R_: 13.7 min

Fig. S3. HPLC profiles of the synthetic peptides.

**pep5**

H-Tyr-(Pro-Hyp-Gly)_4_-Pro-Arg-Gly-Pro-Arg-Gly-Leu-Thr-Gly-Phe-Hyp-Gly-(Pro-Hyp-Gly)_4_-Pro-NH_2_

T_R_: 15.7 min

**pep6**

H-Tyr-(Pro-Hyp-Gly)_4_-Pro-Asn-Gly-Arg-Arg-Gly-Phe-Met-Gly-Met-Hyp-Gly-(Pro-Hyp-Gly)_4_-Pro-NH_2_

T_R_: 16.0 min

**pep7**

H-Tyr-(Pro-Hyp-Gly)_4_-Pro-Lys-Gly-Arg-Arg-Gly-Phe-His-Gly-Leu-Hyp-Gly-(Pro-Hyp-Gly)_4_-Pro-NH_2_

T_R_: 13.1 min

Fig. S3. (*Continued*).

**pep8**

H-Tyr-(Pro-Hyp-Gly)_4_-Pro-Arg-Gly-Pro-Arg-Gly-Leu-Leu-Gly-Leu-Hyp-Gly-(Pro-Hyp-Gly)_4_-Pro-NH_2_

T_R_: 18.7 min

**pep9**

H-Tyr-(Pro-Hyp-Gly)_4_-Pro-Arg-Gly-Phe-Arg-Gly-Leu-Met-Gly-Leu-Hyp-Gly-(Pro-Hyp-Gly)_4_-Pro-NH_2_

T_R_: 19.6 min

**pep10**

H-Tyr-(Pro-Hyp-Gly)_4_-Pro-Arg-Gly-Pro-Arg-Gly-Phe-Thr-Gly-Phe-Hyp-Gly-(Pro-Hyp-Gly)_4_-Pro-NH_2_

T_R_: 16.0 min

Fig. S3. (*Continued*).

**pep11**

H-Tyr-(Pro-Hyp-Gly)_4_-Pro-Val-Gly-Arg-Arg-Gly-Leu-Ser-Gly-Leu-Hyp-Gly-(Pro-Hyp-Gly)_4_-Pro-NH_2_

T_R_: 15.2 min

**pepfreq**

H-Tyr-(Pro-Hyp-Gly)_4_-Pro-Arg-Gly-Arg-Arg-Gly-Leu-His-Gly-Leu-Hyp-Gly-(Pro-Hyp-Gly)_4_-Pro-NH_2_

T_R_: 12.7 min

Fig. S3. (*Continued*).

**pepα1(I)930-938**

**
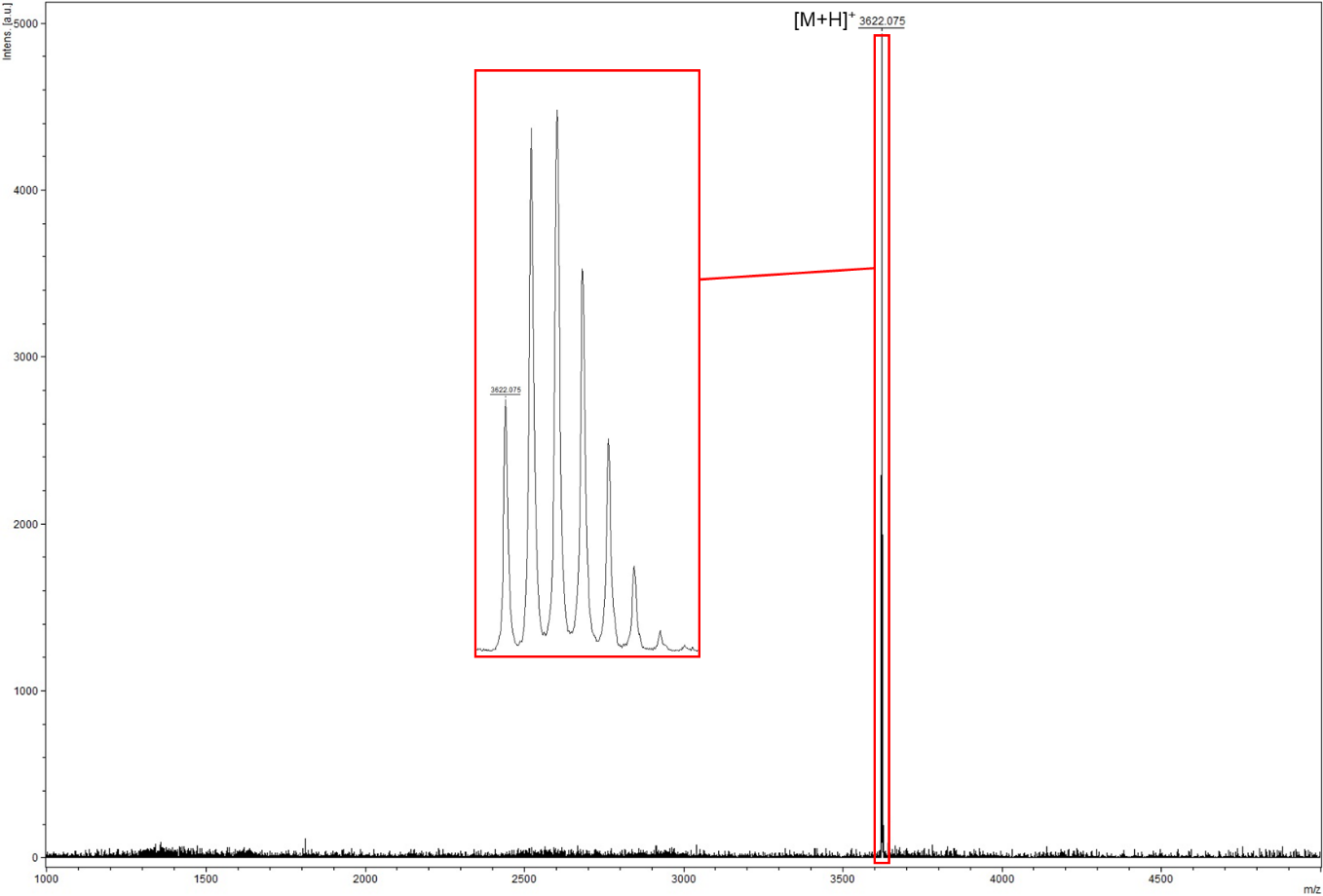
**

calcd. MS [M_m_ + H] ^+^: 3621.75 found: 3622.08

Fig. S4. MS charts of the synthetic peptides.

**pep3**


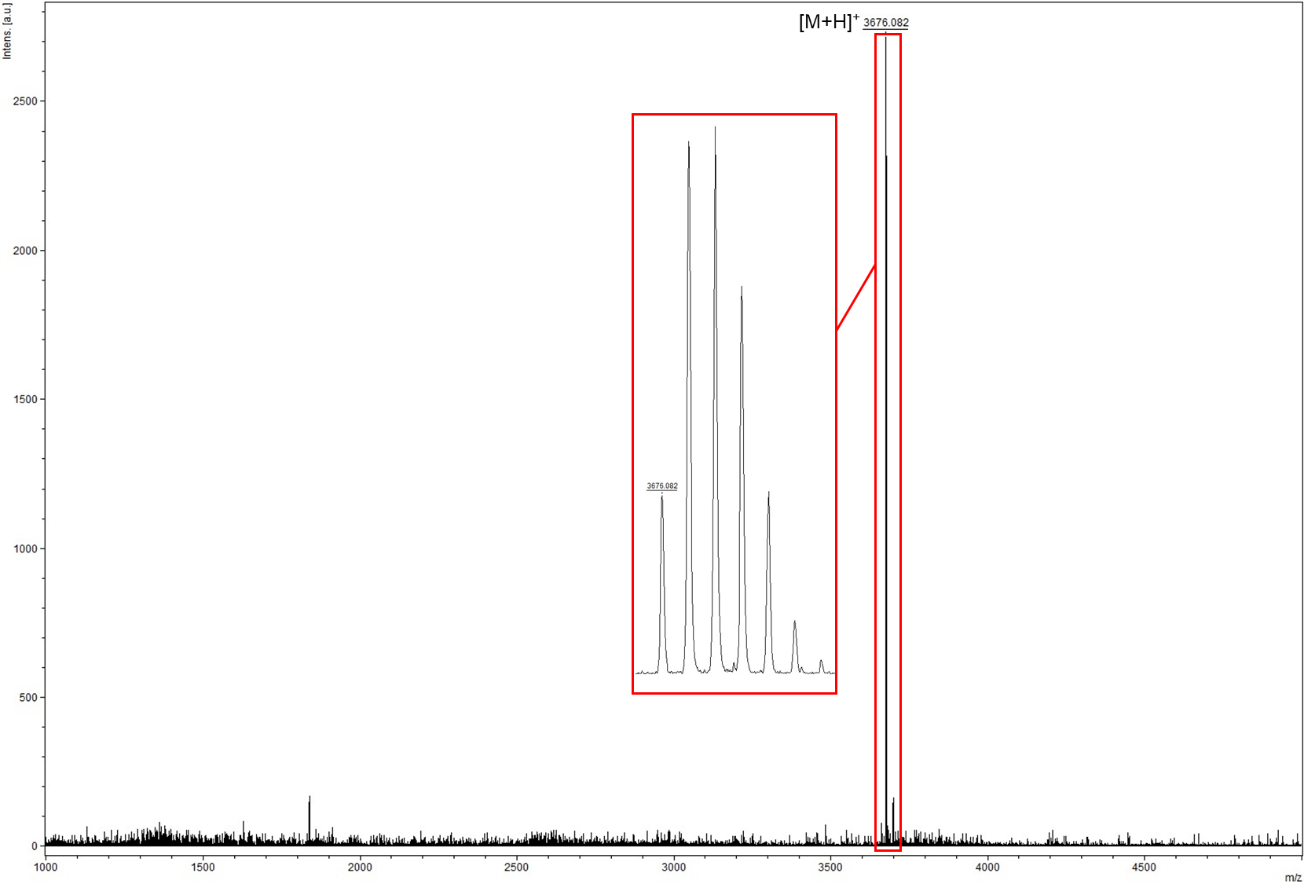


calcd. MS [M_m_ + H]^+^: 3675.81 found: 3676.08

Fig. S4. (*Continued*).

**pep4**

calcd. MS [M_m_ + 3H]^3+^: 1200.60 found: 1200.61

Fig. S4. (*Continued*).

**pep5**

calcd. MS [M_m_ + 3H]^3+^: 1208.59 found: 1208.59

Fig. S4. (*Continued*).

**pep6**

calcd. MS [M_m_ + 3H]^3+^: 1230.24 found: 1230.24

Fig. S4. (*Continued*).

**pep7**

calcd. MS [M_m_ + 3H]^3+^: 1230.95 found: 1230.95

Fig. S4. (*Continued*).

**pep8**

calcd. MS [M_m_ + 3H]^3+^: 1201.28 found: 1201.28

Fig. S4. (*Continued*).

**pep9**

calcd. MS [M_m_ + 3H]^3+^: 1223.94 found: 1223.94

Fig. S4. (*Continued*).

**pep10**

calcd. MS [M_m_ + 3H]^3+^: 1219.92 found: 1219.92

Fig. S4. (*Continued*).

**pep11**

calcd. MS [M_m_ + 3H]^3+^: 1193.27 found: 1193.27

Fig. S4. (*Continued*).

**pepfreq**

calcd. MS [M_m_ + 3H]^3+^: 1228.95 found: 1228.96

Fig. S4. (*Continued*).


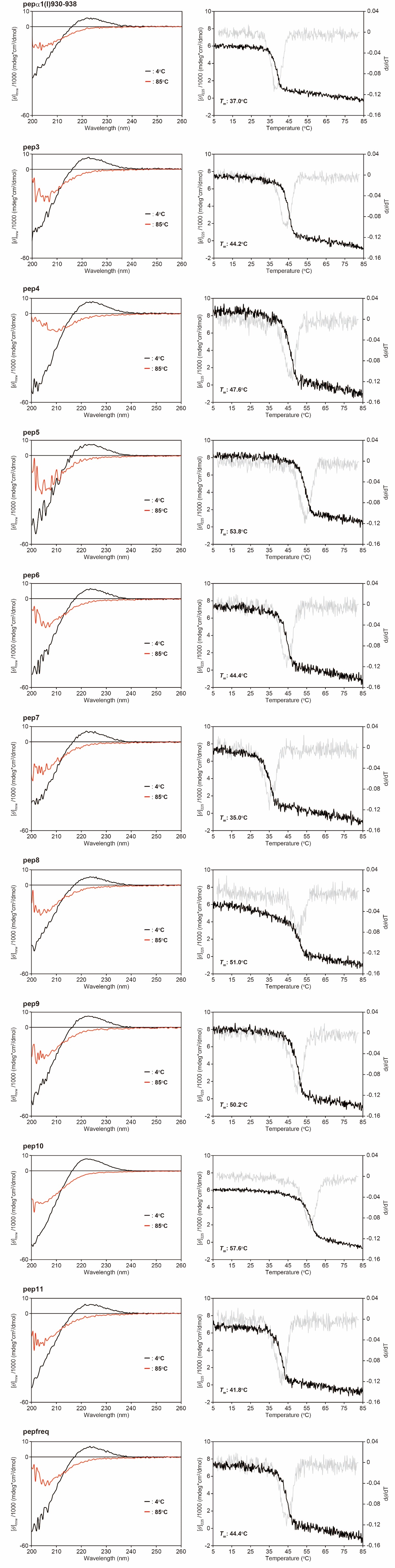


Fig. S5. CD profiles of the synthetic peptides. (Left panels) CD spectrum recorded at 4 and 85°C. (Right panels) A thermal melting curve of the triple helix (black lines) and slope calculated by differentiation (gray lines).


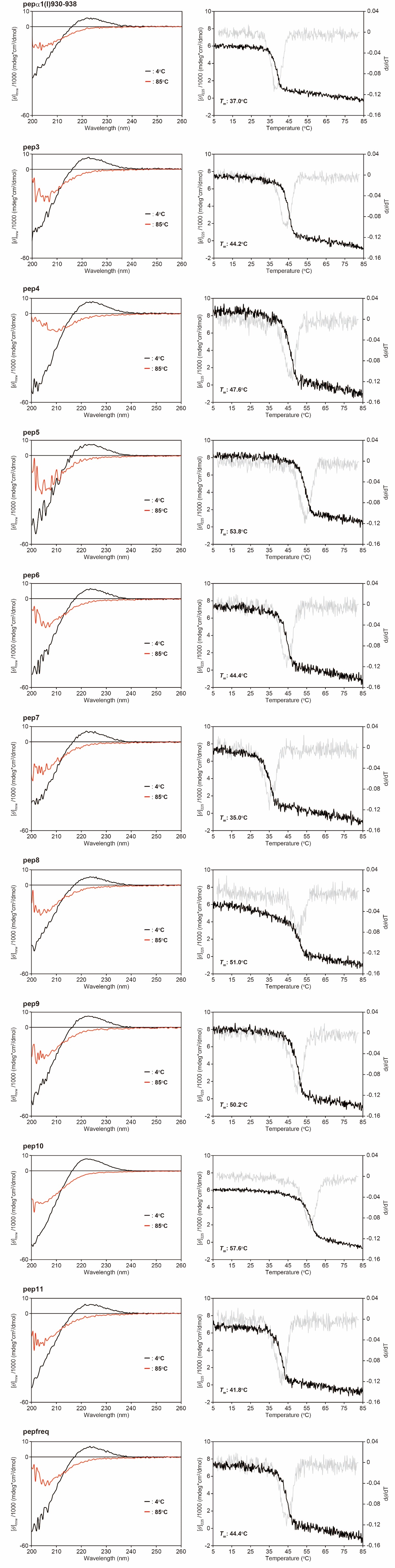


Fig. S5. *(Continued).*


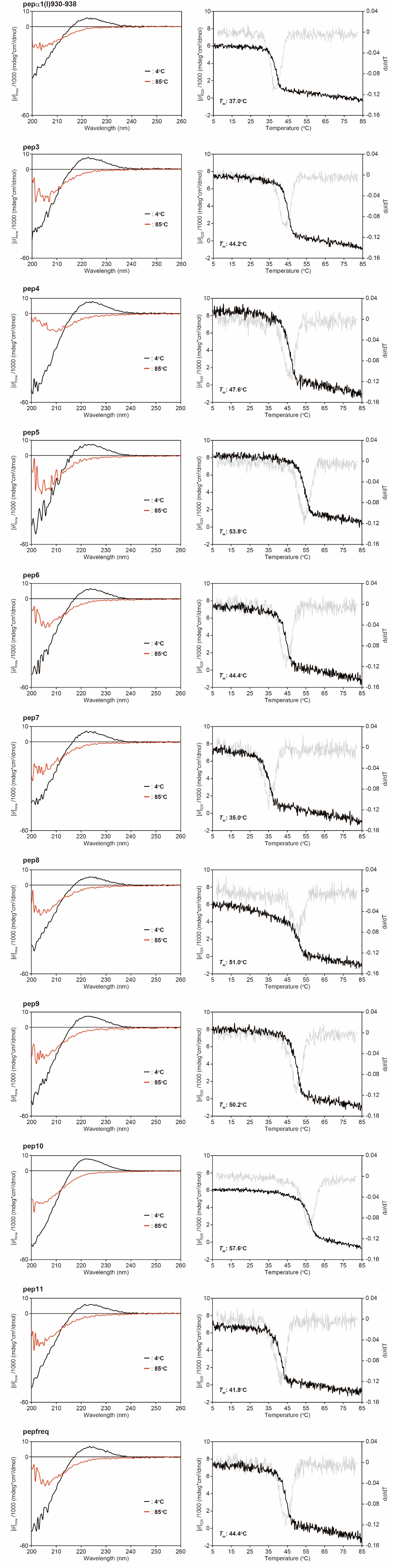


Fig. S5. *(Continued).*

Table S2. Sequences of primers for preparing the bait constructs used in Fig. 4B

| ssDNA | Sequence |
| --- | --- |
| PEDF R148A-forward | 5′-AAACTTGCTGTCAAATCCAGCTTTGTT-3′ |
| PEDF R148A-reverse | 5′-TTTGACAGCAAGTTTCCTCTCAAACAC-3′ |
| PEDF D255N-forward | 5′-GGCTTGAATTCTGATCTCAACTGCAAG-3′ |
| PEDF D255N-reverse | 5′-ATCAGAATTCAAGCCGTATCGTAAGAT-3′ |


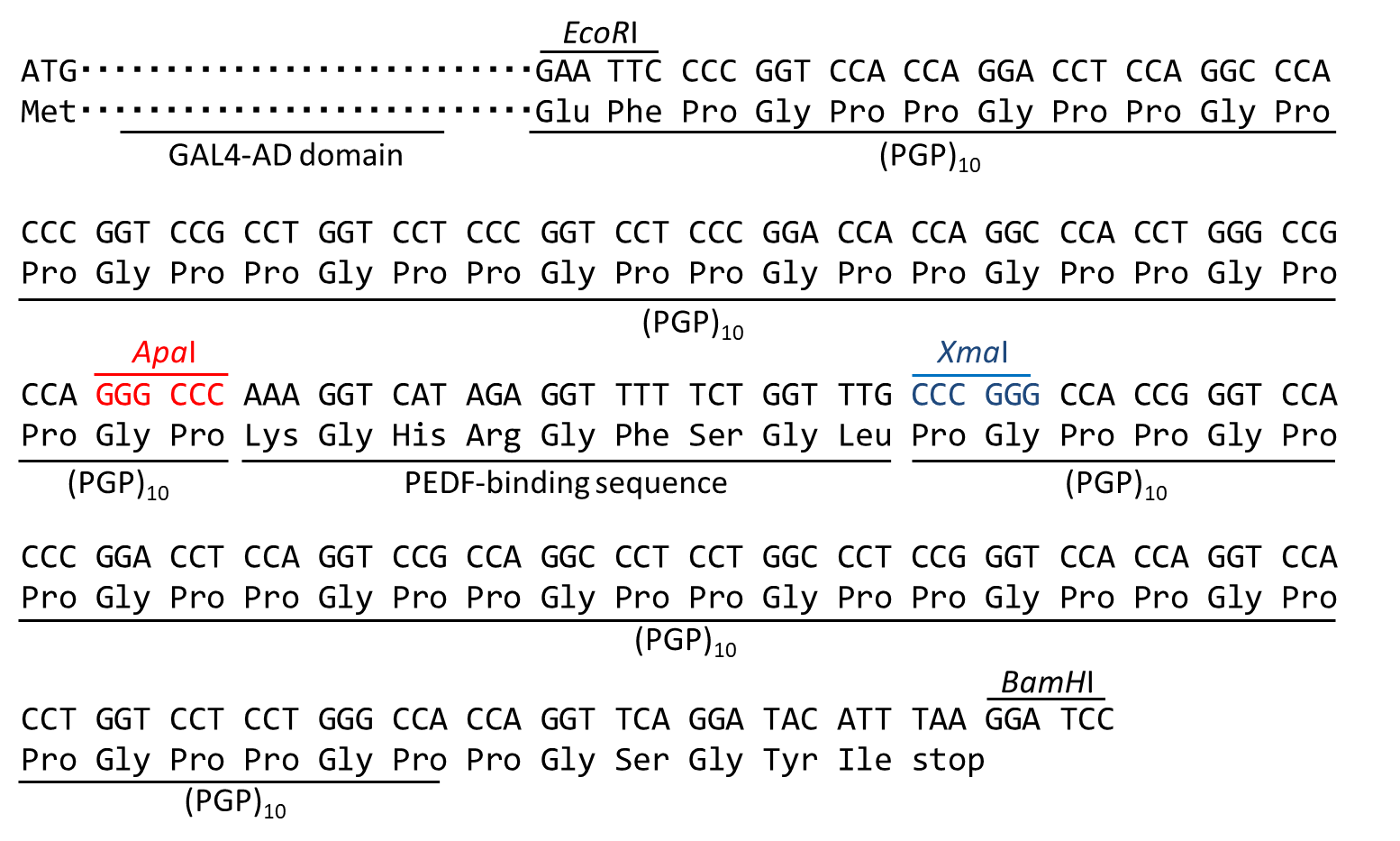


Fig. S6 A sequence map of α1(I)930–938/pGADT7

Table S3. Sequences of ssDNA for preparing the prey constructs used in Fig. 3

| ssDNA | Sequence |
| --- | --- |
| PGP10-sense | 5′-CTGATAGTAATGATAGTAATGATAGTAAC-3′ |
| PGP10-antisense | 5′-CCGGGTTACTATCATTACTATCATTACTATCAGGGCC-3′ |
| PGP13-sense | 5′-CCCAGGTCCTCCAGGTCCTCCAGGTCCTTAGTAAC-3′ |
| PGP13-antisense | 5′-CCGGGTTACTAAGGACCTGGAGGACCTGGAGGACCTGGGGGCC-3′ |
| PGP23-sense | 5′-CCCAGGTCCACCTGGACCACCTGGTCCAC-3′ |
| PGP23-antisense | 5′-CCGGGTGGACCAGGTGGTCCAGGTGGACCTGGGGGCC-3′ |
| 1-1_R4A-sense | 5′-CAAGGGTTGTGCTGGATTGCATGGTCTTC-3′ |
| 1-1_R4A-antisense | 5′-CCGGGAAGACCATGCAATCCAGCACAACCCTTGGGCC-3′ |
| 1-2_R4A-sense | 5′-CAGGGGTGTGGCTGGATTTGAGGGTTGCC-3′ |
| 1-2_R4A-antisense | 5′-CCGGGGCAACCCTCAAATCCAGCCACACCCCTGGGCC-3′ |
| 1-3_R4A-sense | 5′-CCGGGGTCATGCTGGATTTTTGGGTCTTC-3′ |
| 1-3_R4A-antisense | 5′-CCGGGAAGACCCAAAAATCCAGCATGACCCCGGGGCC-3′ |

Table S4. Sequences of primers for NGS samples

| Primer | Sequence |
| --- | --- |
| NGS-forward | 5′-TCGTCGGCAGCGTCAGATGTGTATAAGAGACAGTACCCATACGACGTACCAGATT-3′ |
| NGS-reverse | 5′-GTCTCGTGGGCTCGGAGATGTGTATAAGAGACAGAGATGGTGCACGATGCACAG-3′ |


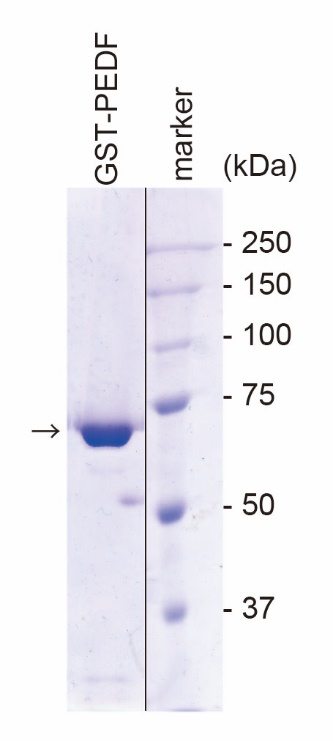


Fig. S7. SDS-PAGE analysis of purified GST-PEDF. Proteins were electrophoresed with 10% acrylamide gel and the bands were visualized by Coomassie Brilliant Blue staining. The molecular sizes are shown in kilodaltons. The molecular weight of GST-PEDF is 72 kDa.

References

1. Sekiya, A., Okano-Kosugi, H., Yamazaki, C. M., Koide, T. (2011) Pigment epithelium-derived factor (PEDF) shares binding sites in collagen with heparin/heparan sulfate proteoglycans. *J. Biol. Chem.* **286**, 26364–26374.
2. Hamaia, S. W., Pugh, N., Raynal, N., Némoz, B., Stone, R., Gullberg, D., *et al.*, (2012) Mapping of potent and specific binding motifs, GLOGEN and GVOGEA, for integrin α1β1 using collagen toolkits II and III. *J. Biol. Chem.* **287**, 26019-26028.
3. Raynal, N., Hamaia, S. W., Siljander, P. RM., Maddox, B., Peachey, A. R., Fernandez, R., *et al.*, (2006) Use of synthetic peptides to locate novel integrin α2β1-binding motifs in human collagen III. *J. Biol. Chem.* **281**, 3821–3831.
4. Hamaia, S. W., Luff, D., Hunter, E. J., Malcor, JD., Bihan, D., Gullberg, D., *et al.*, (2017) Unique charge-dependent constraint on collagen recognition by integrin α10β1. *Matrix Biol.* **59**, 80–94.
5. Zhang, WM., Kapyla, J., Puranen, J. S., Knight, C. G., Tiger, CF., Pentikainen, O. T., *et al.*, (2003) α11β1 integrin recognizes the GFOGER sequence in interstitial collagens. *J. Biol. Chem.* **278**, 7270–7277.
6. Lisman, T., Raynal, N., Groeneveld, D., Maddox, B., Peachey, A. R., Huizinga, E. G., *et al.*, (2006) A single high-afﬁnity binding site for von Willebrand factor in collagen III, identiﬁed using synthetic triple-helical peptides. *Blood.* **108**, 3753–3756 (2006).
7. Giudici, C., Raynal, N., Wiedemann, H., Cabral, W. A., Marini, J. C., Timpl, R., *et al.,* (2008) Mapping of SPARC/BM-40/osteonectin-binding sites on fibrillar collagens. *J. Biol. Chem.* **283**, 19551–19560.
8. Xu, H., Raynal, N., Stathopoulos, S., Myllyharju, J., Farndale, R. W., Leitinger, B. (2011) Collagen binding speciﬁcity of the discoidin domain receptors: Binding sites on collagens II and III and molecular determinants for collagen IV recognition by DDR1. *Matrix Biol.* **30**, 16–26.
9. Konitsiotis, A. D., Raynal, N., Bihan, D., Hohenester, E., Farndale, R. W., Leitinger, B. (2008) Characterization of high afﬁnity binding motifs for the discoidin domain receptor DDR2 in collagen. *J. Biol. Chem.* **283**, 6861–6868.
10. Calvo, E., Tokumasu, F., Mizurini, D. M., McPhie, P., Narum, D. L., Ribeiro, J. M. C., *et al.*, (2010) Aegyptin displays high-affinity for the von Willebrand factor binding site (RGQOGVMGF) in collagen and inhibits carotid thrombus formation in vivo. *FEBS J.* **277**, 413–427.
11. Manka, S. W., Carafoli, F., Visse, R., Bihan, D., Raynal, N., Farndale, R. W., *et al.*, (2012) Structural insights into triple-helical collagen cleavage by matrix metalloproteinase 1. *Proc. Natl. Acad. Sci. U.S.A.* **109**, 12461–12466.
12. Manka, S. W., Bihan, D., Farndale, R. W. (2019) Structural studies of the MMP-3 interaction with triple-helical collagen introduce new roles for the enzyme in tissue remodelling. *Sci. Rep.* **9**, 18785.
